# Cross-reactive immunity against SARS-CoV-2 N protein in Central and West Africa precedes the COVID-19 pandemic

**DOI:** 10.1038/s41598-022-17241-9

**Published:** 2022-07-28

**Authors:** Jannie Pedersen, Ismaël Hervé Koumakpayi, Giorgi Babuadze, Mariana Baz, Oumar Ndiaye, Oumar Faye, Cheikh Tidiane Diagne, Ndongo Dia, Maedeh Naghibosadat, Allison McGeer, Samira Muberaka, Irène P. Moukandja, Stella Ndidi, Carlos B. Tauil, Jean-Bernard Lekana-Douki, Cheikh Loucoubar, Ousmane Faye, Amadou Sall, Kelly G. Magalhães, Nina Weis, Robert Kozak, Gary P. Kobinger, Hugues Fausther-Bovendo

**Affiliations:** 1 Département de Microbiologie-Infectiologie et Immunologie, Université Laval, Quebec City, Canada; 2 Centre Hospitalier Universitaire de Libreville, Libreville, Gabon; 3 Biological Sciences Platform, University of Toronto, Sunnybrook Research Institute at Sunnybrook Health Sciences Centre, Toronto, Canada; 4 Institut Pasteur de Dakar, Dakar, Senegal; 5 Department of Laboratory Medicine and Pathobiology, University of Toronto, Toronto, Canada; 6 Department of Microbiology, Sinai Health System/University Health Network, Toronto, Canada; 7 Department of Laboratory Medicine and Molecular Diagnostics, Division of Microbiology, Sunnybrook Health Sciences Centre, Toronto, Canada; 8 Laboratory of Immunology and Inflammation, University of Brasilia, Brasilia, Brazil; 9 Unité d’Evolution Epidémiologie et Résistances Parasitaires, Centre Interdisciplinaire de Recherches Médicales de Franceville, Franceville, Gabon; 10 Department of Infectious Diseases, Copenhagen University Hospital, Hvidovre, Denmark; 11 Department of Clinical Medicine, Faculty of Health and Medical Sciences, University of Copenhagen, Copenhagen, Denmark; 12 Galveston National Laboratory, University of Texas Medical Branch, Galveston, TX USA; 13 Global Urgent and Advanced Research and Development, 911 Rue Principale, Unit 100, Batiscan, QC G0X 1A0 Canada

**Keywords:** Infectious-disease diagnostics, Virology, Viral infection

## Abstract

Early predictions forecasted large numbers of severe acute respiratory syndrome coronavirus (SARS-CoV-2) cases and associated deaths in Africa. To date, Africa has been relatively spared. Various hypotheses were postulated to explain the lower than anticipated impact on public health in Africa. However, the contribution of pre-existing immunity is yet to be investigated. In this study, the presence of antibodies against SARS-CoV-2 spike (S) and nucleocapsid (N) proteins in pre-pandemic samples from Africa, Europe, South and North America was examined by ELISA. The protective efficacy of N specific antibodies isolated from Central African donors was tested by in vitro neutralization and in a mouse model of SARS-CoV-2 infection. Antibodies against SARS-CoV-2 S and N proteins were rare in all populations except in Gabon and Senegal where N specific antibodies were prevalent. However, these antibodies failed to neutralize the virus either in vitro or in vivo. Overall, this study indicates that cross-reactive immunity against SARS-CoV-2 N protein was present in Africa prior to the pandemic. However, this pre-existing humoral immunity does not impact viral fitness in rodents suggesting that other human immune defense mechanisms could be involved. In Africa, seroprevalence studies using the N protein are over-estimating SARS-CoV-2 circulation.

## Introduction

The novel coronavirus, denoted severe acute respiratory syndrome (SARS) coronavirus (CoV) 2 (SARS-CoV-2), is the causative agent for the current coronavirus disease 2019 (COVID-19) pandemic^1^. Since December 2019, SARS-CoV-2 has rapidly spread throughout the globe. As of July 2021, more than 249 million COVID-19 cases have been recorded worldwide, resulting in more than 3.9 million deaths. Surprisingly, only 151,000 of the 5 million COVID-19 related deaths have been reported on the African continent and, of those, the great majority (89,319; 59%) have occurred in South Africa. In contrast, South America and notably Brazil have been severely affected by the current pandemic. More than 21 millions SARS-CoV-2 infections and 609,000 associated deaths have been reported in Brazil (https://covid19.who.int/, https://coronavirus.jhu.edu/map.html). The factors influencing the disparity in the spread of SARS-CoV-2 in various countries remain poorly understood.

Due to the weak health system of a large number of African countries, initial models projected COVID-19 caseload and associated mortality of much higher magnitude on the African continent^2^. Since then, various factors have been postulated to have contributed to the unexpectedly low numbers of reported cases of COVID-19 on the continent including high prevalence of mild symptomatic disease in a younger population and under-reporting associated with limited testing and health care capacity. Several seroprevalence studies conducted in African countries have suggested higher SARS-CoV-2 prevalence than expected based on confirmed case counts^3,4^. However, the initial delay in the emergence of the first COVID-19 cases, the strict quarantine/restriction measures put in place by African countries and lessons learned from previous outbreaks of emerging pathogens may have all contributed to the low number of COVID-19 cases reported in Africa^2,5^. Additional factors may also be at play.

In addition to SARS-CoV-2, six coronaviruses are known to infect humans. Severe acute respiratory syndrome coronavirus (SARS-CoV) caused a human outbreak between 2002 and 2003, while since 2012, frequent spill-over events of Middle East respiratory syndrome coronavirus (MERS-CoV) have led to sporadic human outbreaks in the Middle East. In contrast, human coronavirus (HCoV) 229E, OC43, NL-63 and HKU1 circulate worldwide in the human population (reviewed in^6^). Coronaviruses are also highly prevalent in wild animals, notably in bats; viral nucleic acids have been detected from bats in Africa, Asia, America and Europe^7^.

During SARS-CoV-2 infections, the nucleocapsid (N) and spike (S) proteins are immune-dominant, with the great majority of the antibodies targeting these antigens^8^. Among them, the N protein is highly conserved between coronavirus infected humans and animals, while the S protein is more genetically diverse^6,9^.

This study documents the presence of antibodies against SARS-CoV-2 N and S proteins in pre-pandemic sera (collected prior to November 2019) from individuals from various continents as well as the in vitro neutralization capacity and the in vivo protective efficacy of some of the sera with high antibody levels against SARS-CoV-2 N protein.

## Results

### Prevalence of antibodies against SARS-CoV-2 N protein in the Gabonese population (Central Africa)

We hypothesized that cross-protective immunity against SARS-CoV-2 was present in a large proportion of the population of Africa prior to the start of the current COVID-19 pandemic. To investigate this hypothesis, IgG antibody level against bovine serum albumin (BSA) and two SARS-CoV-2 proteins, its spike (S) and nucleocapsid (N), were simultaneously measured using in-house serological assays. Serum from 43, 121, 112 and 146 individuals from Quebec (Canada), Denmark, Brazil and Gabon (Central Africa) were tested. All sera were collected before November 2019, prior to the start of the COVID-19 pandemic (Table 1). Convalescent sera from 12 laboratory confirmed COVID-19 cases in Ontario (Canada) were used as positive control.

**Table 1:** Study cohort.

Country	Sample size	Sampling time period	Age (years)
Canada (confirmed)	12	Mars 2020–Apr 2020	50–70
Canada (pre-pandemic)	43	Feb 2018–Nov 2019	> 18*
Brazil	112	Feb 2016–Dec 2017	> 18*
Denmark	121 (118)	Apr 2007–Dec 2019	> 18*
Gabon	146	Oct 2019	18–50*
Senegal (confirmed)	2	Apr 2021	69–74
Senegal (pre-pandemic)	150	Jan 2017–May 2018	0–75

The size, collection period and age is indicated for the various samples cohort used in this study. *Codified samples from adult donors (> 18 years old) were obtained. No donors specific information were available as indicated in the respective ethic protocols.

First, IgG levels against BSA were analyzed. 2/43, 4/121, 4/118, 30/146 and 1/12 samples from the Canada, Denmark, Brazil, Gabon and COVID-19 confirmed cases groups were positive for BSA-specific IgG. These samples had OD above 0.44, which corresponded to the mean plus 3 standard deviations (SD) of the Denmark group (Fig. S1). These samples were excluded from the subsequent analysis to prevent the inclusion of non-specific signals.

**Figure 1:**
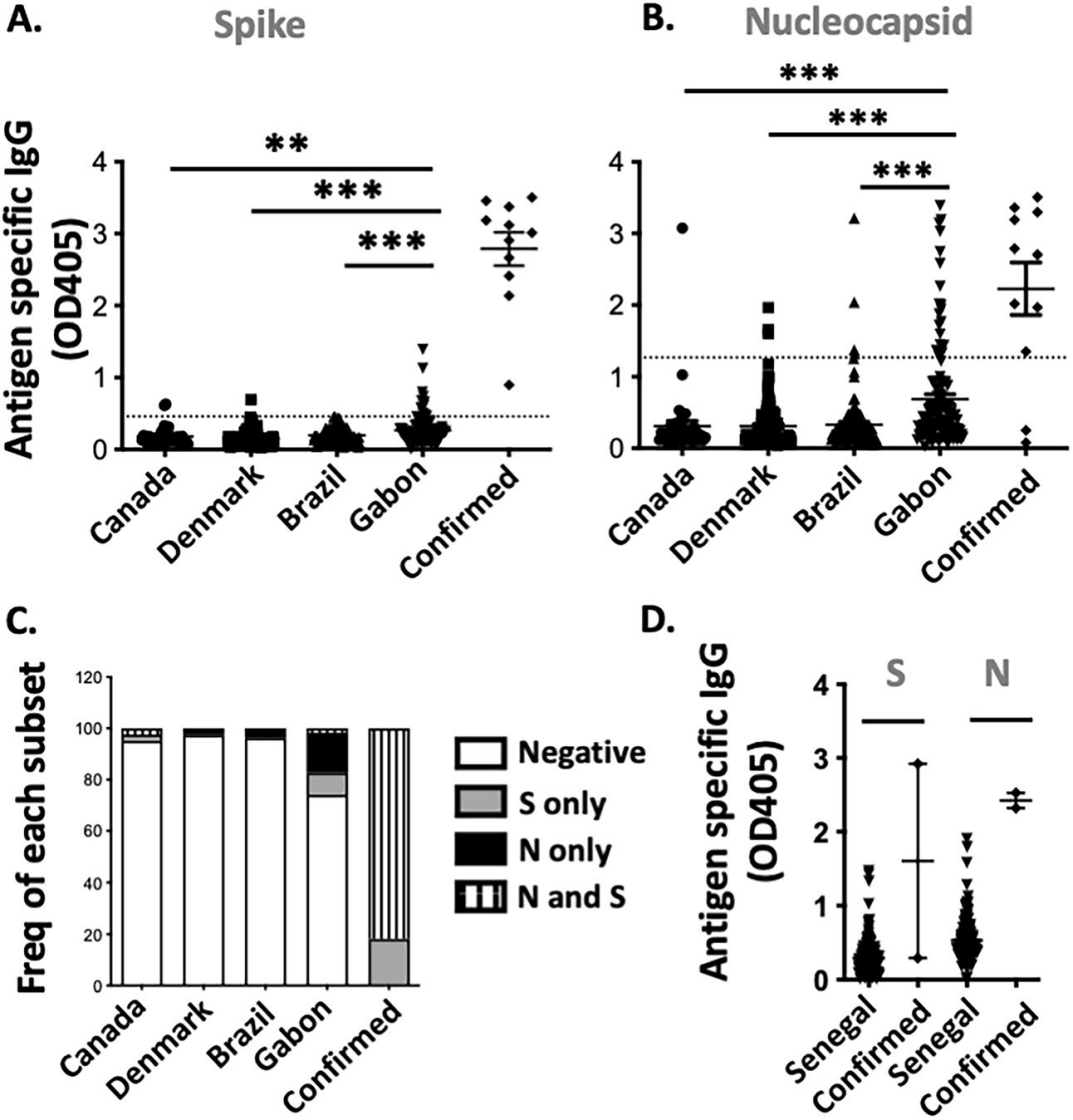
Pre-existing immunity against SARS-CoV-2 S and N protein in various populations. Serological screening for the presence of IgG against SARS-CoV-2 S (**A**,**D**) and N protein (**B**,**D**). Sera from Quebec (n = 41), Denmark (n = 117), Brazil (n = 108) and Gabon (n = 116), and 11 COVID-19 confirmed cases (**A**,**B**) as well as pre-pandemic (n = 128), and COVID-19 confirmed sera (n = 2), from Senegal (**D**) were analyzed by ELISA. The proportion of individuals with antibodies against either or both the SARS-CoV-2 S and N proteins is illustrated (**C**).

The humoral response against SARS-CoV-2 N and S proteins was subsequently studied. All laboratory confirmed COVID-19 cases had a detectable level of IgG against the S proteins. In contrast, S specific IgG were rare or undetectable in all sera from Canada, Denmark and Brazil. In the Gabon group, 12/116 (10.3%) of the samples were positive. However, the level of antibody against the S protein were generally low (Fig. 1A). High levels of N specific IgG were detectable in 9/11 (81.8%) of COVID-19 confirmed cases as well as 1/41 (2.4%), 3/121 (2.5%); 4/108 (3.7%) and 20/116 (17.2%) of sera from Canada, Denmark, Brazil and Gabon respectively. Of note, the magnitude of IgG specific response against the N protein was significantly higher (p < 0.001) in the Gabon group compared with samples from Canada, Denmark and Brazil (Fig. 1B). Interestingly, unlike in SARS-COV-2 confirmed cases, in sera from Gabonese donors, humoral responses were detected against either S or N and rarely concomitantly (Fig. 1C, Fig. S2).

To investigate whether pre-pandemic cross-reactive humoral responses against SARS-COV-2 were restricted to Gabon, sera from 150 individuals from Senegal (West Africa) collected prior to May 2018 were analysed. Samples from 2 laboratory confirmed cases from Senegal were also included. Of note, unlike the previous samples, sera from Senegal were tested locally. Samples were tested for reactivity to BSA, SARS-CoV-2 N and S proteins (Fig. S1). Samples reactive to BSA (n = 6) were excluded from subsequent analysis (Fig. S1). In pre-pandemic samples from Senegal, both S and N specific IgG were detected in a large proportion of tested samples (Fig. 1D). In samples from Senegal, the levels of IgG against SARS-CoV-2 S and N correlated (p value < 0.001). As noted above, in samples from Gabon, no such correlation was observed (p value 0.23) (Fig. S2). This might suggest that cross-reactive humoral responses against SARS-CoV-2 originate from distinct mechanisms in Senegal and Gabon. Alternatively, the difference in the observed humoral response may be due to the age of the donors from Senegal. They were much younger than the other cohorts, with 60/150 (40%) and 38/150 (25.3%) of donors aged between 0–9 and 10–17 year old respectively.

### Lack of protective efficacy in rodents from polyclonal antibodies against SARS-CoV-2 N protein

**Figure 2:**
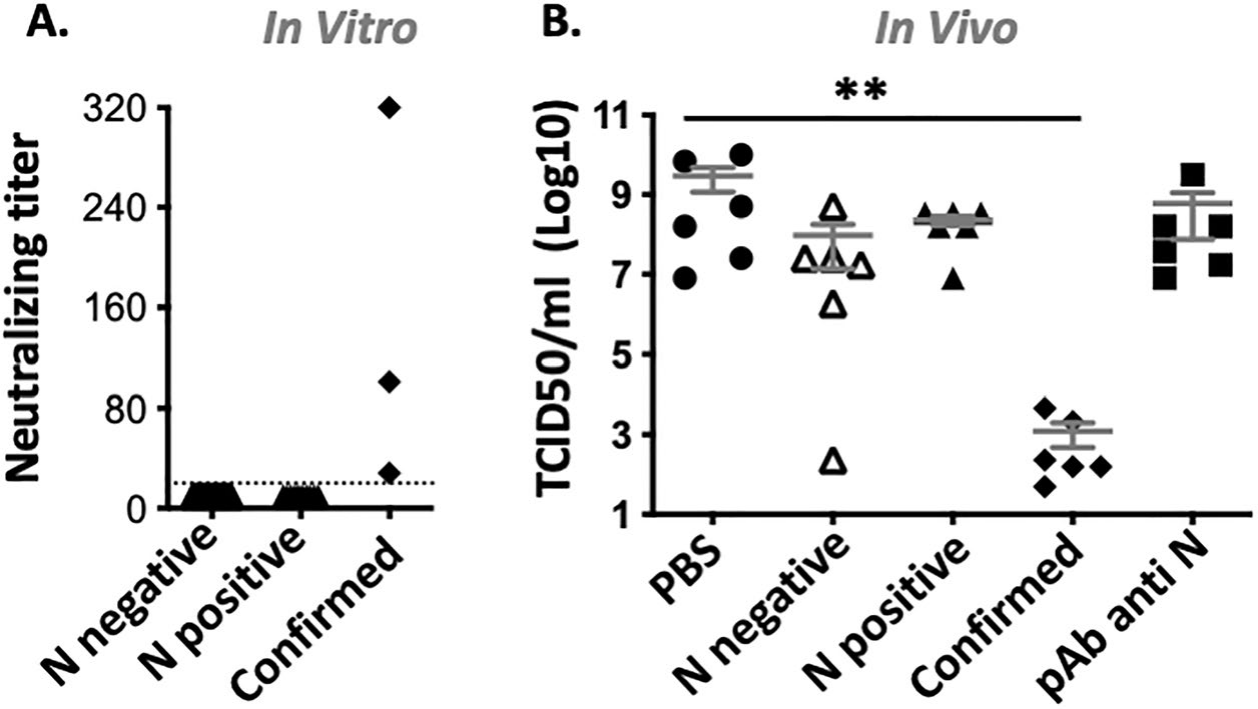
In vitro and in vivo neutralization of human sera with antibodies against SARS-CoV-2 N protein. (**A**) Neutralising titer of sera from Gabonese donors with (N positive) or without (N negative) Ab against SARS-CoV-2 N protein (n = 10/group). Standard sera from COVID-19 confirmed cases with high, medium and low neutralization capacity were used as positive control. Sera with neutralizing titer below the lowest tested dilution (dotted line), are plotted at 10. (**B**) Day 5 lung viral titer (TCID50/ml) in SARS-CoV-2 challenged mice (n = 6/group) pretreated with either PBS; 1 mg purified IgG from Gabonese sera with (N positive) or without (N negative) Ab against SARS-CoV-2 N protein; or from COVID-19 confirmed cases (confirmed) or with 50 μg polyclonal antibodies against SARS-CoV-2 N (pAb anti N). Mean ± SEM is depicted.

To determine whether the antibodies against N observed in the Gabonese population could be protective against severe disease, the in vitro neutralization capacity and the in vivo protective efficacy of these sera were tested. For in vitro neutralization, 20 sera from Gabon, half with and the other half without antibodies against SARS-CoV-2 N protein were tested. Of note, none of the selected sera had detectable antibodies against SARS-CoV-2 S protein. Sera from 3 COVID-19 confirmed cases were used as positive controls. Neutralization, at titers ranging from 320 to 28, was observed in COVID-19 confirmed cases. No neutralization (titer < 20), was observed in any sera from Gabon independently of the presence or absence of antibodies against SARS-CoV-2 N protein (Fig. 2A).

Next, the ability of antibodies from Gabonese samples to influence viral load was monitored in the mouse model of SARS-CoV-2 infection. To do so, total IgG was purified using the protein G HP SpinTrap from sera from Gabonese donors with or without antibodies against SARS-CoV-2 N protein, as well as COVID-19 confirmed cases (6–8 donors/group). All sera from Gabon were devoid of antibody against SARS-CoV-2 S protein. Human ACE2 transgenic mice received intraperitoneally 1 mg each of purified IgG from 1 or 2 donors from the same group. The purified antibodies from survivors were used as a positive control as well as mice receiving 50 μg of a commercial polyclonal antibody against SARS-CoV-2 N protein. Mock treated mice were used as negative control. Mice were euthanized 5 days post infection, and infectious lung viral titers were determined by TCID50. Lung viral load decreased from 1.8 × 10^10^ TCID50/ml in mock treated mice to 7.4 × 10^3^ TCID50/ml (p < 0.01) in mice treated with purified IgG from SARS-CoV-2 confirmed cases (Fig. 2B). In contrast, no difference in lung viral titer were observed between mock treated mice, mice that received purified IgG from Gabonese donors with and without anti-SARS-CoV-2 N Ab and mice treated with commercial anti N antibodies, (mean TCID50/ml of 1.8 × 10^10^, 1.4 × 10^9^, 5.8 × 10^8^ and 3.6 × 10^9^ respectively, p > 0.05) (Fig. 2B).

## Discussion

Overall, under our experimental conditions, cross-reactive antibodies against SARS-CoV-2 N protein do not significantly reduce the viral load in the lungs. Whether the presence of these antibodies correlates with decreased pathology or moderation of symptoms remains to be investigated. In the mouse model of infection, the Fc mediated antibody functions of the purified human antibodies are limited, hence underestimating their therapeutic contributions^10^. Furthermore, our work is limited to the humoral response against SARS-CoV-2. Individuals from Gabon and Senegal likely also have cross-reactive T cells against SARS-CoV-2 N protein. Upon exposure to SARS-CoV-2, these individuals would mount a recall response resulting in a sharp increase in the magnitude of both cellular and humoral response. Therefore, our results from the mouse model of infection probably underestimate the protective efficacy of cross-reactive immunity against SARS-CoV-2 N protein. It is worth noting that recent studies have demonstrated the protective role of the adaptive immunity against the SARS-CoV-2 N protein. First, immunizing mice with both SARS-CoV-2 N and S proteins improved vaccine efficacy, notably viral control within the brain of SARS-CoV-2 challenged mice^11^. Second, a peptide vaccine encoding for T cells epitopes from the N protein was able to prevent lung abnormalities and reduce viral shedding in the majority of non-human primates challenged with SARS-CoV-2^12^. Finally, a N specific antibody, isolated from a recovering individuals, was demonstrated to inhibit SARS-CoV-2 N mediated complement hyperactivation. The latter is a risk factor for morbidity and mortality in SARS-CoV-2 infected individuals^13^. The above studies suggest that N specific T cells and antibodies can both impact the severity of COVID-19.

Cross-reactive cellular and humoral immunity against additional SARS-CoV-2 protein may also contribute to SARS-CoV-2 protection. It is worth noting that younger demographic alone cannot explain the lower number of reported COVID-19 cases in Africa. Several countries in South America and Asia, have young populations but still reported significantly larger SARS-CoV-2 circulation compared with African countries.

Coronaviruses circulate widely in both the animal and human populations^6^. Spill-over events from susceptible animals to individuals can lead to human exposures. SARS-CoV-2 is closely related to the bat coronavirus, Bat Cov RatG13. The current COVID-19 pandemic may therefore have originated from a spill-over event from an infected bat^1^. Similar spill-over events of less pathogenic coronaviruses into the human populations may have occurred previously, or maybe occurring on an ongoing basis.

Individuals from Gabon and Senegal exhibited high levels of antibodies against the SARS-CoV-2 N and/or S protein. In contrast, individuals from the other tested locations, including Brazil, Canada and Denmark, had no or few antibodies against either protein. This low level of cross-reactive antibodies against SARS-CoV-2 are in line with previous studies. Low levels of pre-existing humoral against the SARS-CoV-2 S protein in uninfected individuals has also been documented in France, England and the US^14–16^.

The underlying causes for the high seropositivity against SARS-CoV-2 in the Gabonese and Senegalese populations remain elusive. The common HCoVs, 229E, OC43, NL-63 and HKU1, are ubiquitous throughout the globe^6^. Others have hypothesized that infections with these common HCoVs could lead to cross-reactive antibodies against SARS-CoV-2^17,18^. However, a recent serological study demonstrated that antibodies against the N protein of common HCoVs are equally present in Africa, Europe and South Africa and their presence do not correlate with cross-reactive antibodies against SARS-CoV-2 N protein^18^. It is therefore highly unlikely that common HCoVs are the main contributor to the higher cross-reactive immunity observed in sera from Gabon and Senegal. In these pre-pandemic samples, high seropositivity against SARS-CoV-2 N could be indicative of the undocumented circulation of other as yet unrecognized coronaviruses. While the S proteins of coronaviruses are more diverse, their N proteins are far more conserved^6,9^. At the amino acid level, SARS-CoV-2 N protein shows between 93 and 100% homology with the N protein of several coronaviruses from bats (QHR63308.1; AVP78038.1; AVP78049.1) and pangolin origin (including QIG55953.1, QIA48630.1, QIQ54056.1, QIA48648.1) (Fig. S2). Both animals are present in Central and West Africa; and several coronaviruses from both the alpha and betacoronavirus genus, have been isolated from bats in Gabon and Guinea^19,20^. These coronaviruses were however distinct from SARS-CoV-2. It is also worth noting that the possibility of different pathogens able to induce cross-reactive antibodies to SARS-CoV-2 N protein cannot be excluded. However, the co-occurrence of antibodies against SARS-CoV-2 and other pathogens is not sufficient to conclude that these pathogens are responsible for cross-reactive antibodies, especially in areas endemic for *Plasmodium* and Dengue virus^21^.

The above findings are restricted to Gabon and Senegal. Of note, separate studies also found high pre-existing immunity against SARS-CoV-2 N and to a lower extend S protein in samples from Nigeria, Ghana, Benin (West Africa), Gabon (Central Africa), as well as Tanzania (East Africa) and Zambia (Southern Africa) indicating that this phenomenon is wide spread in Africa^17,18,22,23^. Existing serological assays relying on the detection of antibodies against either SARS-CoV-2 N or S protein. These assays, especially the ones monitoring SARS-CoV-2 N antibodies, will overestimate SARS-CoV-2 prevalence in Africa due to the significant number of false positives. For accurate evaluation of SARS-CoV-2 seroprevalence in Africa, the specificity of the serological assays used should first be confirmed using local pre-pandemic samples. Furthermore, a high proportion of BSA reactive samples were detected in Gabon and Senegal. Such samples exhibiting non-specific binding should also be excluded from seroprevalence studies.

Overall, the evidence presented herein supports the idea that cross-reactive humoral immunity against SARS-CoV-2 N protein is common in Gabon and Senegal. Individually, antibodies targeting SARS-CoV-2 N protein did not impact viral load in the mouse model of infection, in which Fc mediated functions are not fully recapitulated and which does not take into account the sharp rise in adaptive immunity following a recall response. Additional work will be required to determine whether the presence of both cellular and humoral responses against the SARS-CoV-2 N protein could contribute to the lower COVID-19 disease prevalence observed in Africa.

## Methods

### Study approval

Following patient consent, sera from COVID-19 confirmed cases were collected from patients in Toronto. The above sample collection was approved by the Sunnybrook Health Sciences research ethic board under the protocol #2218. The use of retrospective samples did not require individual patient consent. Requirement for inform consent was waived by the National ethical committees of Gabon (N° 003/2020/CNER/SG/P), the ethical committees at Université Laval (Canada, 2020-5142), the Copenhagen University Hospital (Denmark, H-20083193) and the University of Brasilia (Brazil, 36249214.0.0000.5553) which all approved this study. COVID-19 sera samples from the Pasteur Institute of Dakar (Senegal) were collected from the WHO reference laboratory for flu and only anonymized samples were used retrospectively in this study. Requirement for inform consent for samples from Senegal was waived by the ethical committee of the Pasteur Institute of Dakar. All human experiments were performed according to the relevant guidelines and regulations of the different countries involved.

Animal experiments were approved by the institutional review board (animal committee) of the University of Toronto (APR-00005433-v0002-0) and conducted according to the guidelines of the Canadian Council on Animal Care. Mice were acclimatized for a week before the experiment and monitored daily thereafter. Infectious work was performed in the biosafety level 3 laboratory at the University of Toronto or at Université Laval. The study was carried out in compliance with the ARRIVE guidelines.

### ELISA

Individual wells were coated overnight at 4 °C with 50 ng of BSA (Thermofisher Scientific, Burlington, Canada), SARS-CoV-2 nucleocapsid (#40588-V08B, Cedarlane labs, Burlington, Canada) or SARS-CoV-2 spike protein, kindly provided by Yannick Doyon. After 6 washes, wells were blocked with PBS, 5% milk for an hour. Wells were then incubated with 1/100 diluted sera for an additional hour at 37 °C. Following extensive washes, wells were incubated for an hour with 15 ng of HRP conjugated goat anti human IgG (Thermofisher Scientific). After a final round of washes, well were incubated with 50ul of ABTS substrate (Mandel Scientific, Guelph, Canada). The absorbance was then read at 405 nm.

### In vitro microneutralization

The microneutralization assay was adapted from an in-house assay used for pathogenic avian influenza viruses^24,25^. Briefly, serial twofold dilutions of heat-inactivated serum (30 min at 56 °C) were prepared starting from a 1:20 dilution. Equal volumes of serum and virus were mixed and incubated for 60 min at room temperature. The residual infectivity of the virus-serum mixture was determined in Vero E6 cells (ATCC, Manassas, VA) using four wells for each serum dilution. Neutralizing antibody titer was defined as the reciprocal of the serum dilution that completely neutralized the infectivity of 100 TCID50 of SARS-CoV-2 (SARS-CoV-2/Quebec City/21697/2020) as determined by the absence of cytopathic effect (CPE) on Vero cells at day 4.

### SARS-CoV-2 mice challenge

Female 6–8-week-old K18-ACE2 transgenic mice were purchased from Jackson Lab (Bar Habor, ME). Mice were acclimatized for 1 week in the University of Toronto’s BSL3 facility prior to the start of the experiment. Animals (n = 6 per group) were injected intraperitoneally with DPBS, 1 mg of purified IgG from sera from Gabon or COVID-19 confirmed cases, or 50 μg of anti SARS-CoV nucleocapsid (ABIN129544, antibodies-online.com, Limerick, PA). Mice were then challenge intranasally with 4.2 × 10^4^ TCID50 passage 3 of SARS-CoV-2/SB3-TYAGNC^26,27^. Animals were euthanized, by cardiac puncture, 5 days post-challenge. Lung viral load (TCID50) was determined. Briefly, serially diluted lung homogenates from individual mice were added to Vero E6 cells monolayer for an hour at 37 °C, then replaced with media supplemented with 2% FBS. Cells were incubated at 37 °C. After 5 days, each well was scored for the presence or absence of CPE. The Spearman Kärber formula was then used to calculate TCID_50_/mL.

### Statistical analysis

The humoral response (OD405) and TCID50 from the various groups were compared by One-Way ANOVA followed by the Dunnett’s test. “**”, “***” are indicative of p values inferior to 0.01 and 0.001 respectively. For ELISA, samples were considered positive if their OD was above the mean plus 3 standard deviations of the Denmark group, for which a large number of sample were available.

## Supplementary Information

Supplementary Figure S1.

Supplementary Figure S2.

Supplementary Figure S3.

Supplementary Legends.
