# Supplementary figures and images for: Cross-reactive immunity against SARS-CoV-2 N protein in Central and West Africa precedes the COVID-19 pandemic
Source: Sci Rep. 2022 Jul 28;12:12962. doi: 10.1038/s41598-022-17241-9 (PMC9333058; doi:10.1038/s41598-022-17241-9)

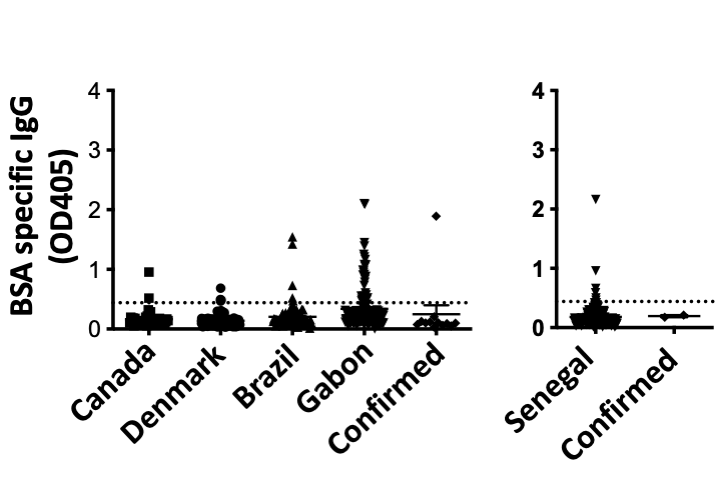

Supplement: Supplementary file 1 — Supplementary Figure S1. [file 41598_2022_17241_MOESM1_ESM.tiff]

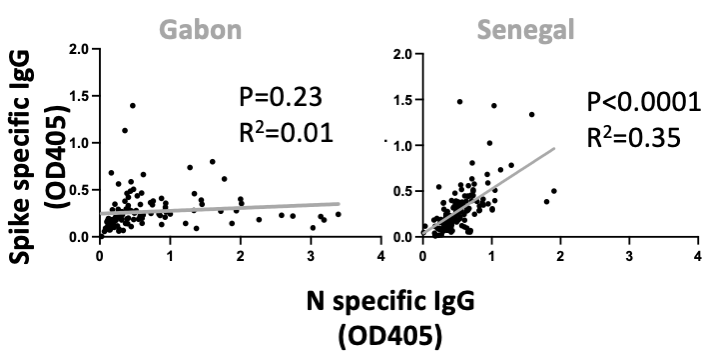

Supplement: Supplementary file 2 — Supplementary Figure S2. [file 41598_2022_17241_MOESM2_ESM.tiff]

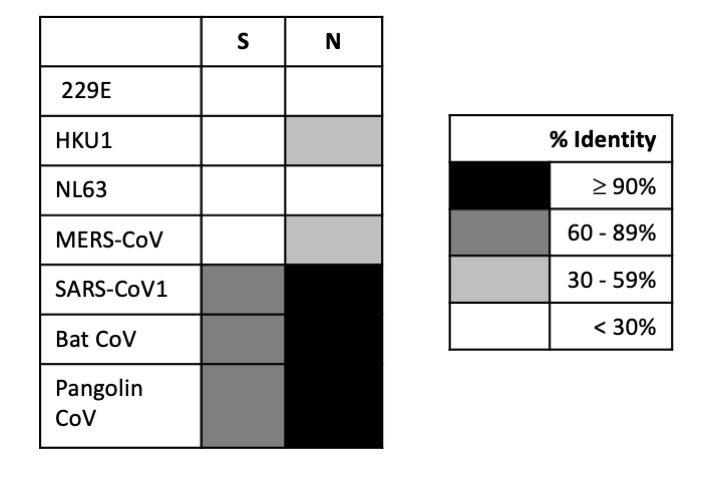

Supplement: Supplementary file 3 — Supplementary Figure S3. [file 41598_2022_17241_MOESM3_ESM.tiff]
