# Supplementary material for: Cross-reactive immunity against SARS-CoV-2 N protein in Central and West Africa precedes the COVID-19 pandemic
Source: Sci Rep. 2022 Jul 28;12:12962. doi: 10.1038/s41598-022-17241-9 (PMC9333058; doi:10.1038/s41598-022-17241-9)
Supplement: Supplementary file 4 — Supplementary Legends. [file 41598_2022_17241_MOESM4_ESM.docx]

**Supplemental Material**

**Figure S1**: **Non-specific IgG Levels in sera from the different groups.**

Samples were tested for binding to BSA to excluded sera non-specific binding in subsequent serological assays. Samples from Canada (n=43), Denmark (n=121), Brazil (n=112), Gabon (n=146), COVID-19 confirmed cases (n=12) (**left**) as well as sera from COVID confirmed cases (n=2) and pre-pandemic samples (n=153) from Senegal (**right**) were screened by ELISA.

**Figure S2: Correlation between IgG response against SARS-CoV-2 S and N proteins.**

The levels of antibodies against SARS-CoV-2 S and N proteins are illustrated for pre-pandemic samples from Gabon (n=146) and Senegal (n=153). R^2^ and p values for the linear curves are indicated.

**Figure S3: Sequence homology between various coronavirus.**

Percentage homology between the spike (S) and nucleocapsid (N) protein of SARS-CoV-2, and endemic coronavirus as well as bat and pangolin coronavirus are indicated. represent ≥ 90, 60-89, 30 -59, and < 30% homology respectively.
